# Cohesin-dependent chromosome loop extrusion is limited by transcription and stalled replication forks

**DOI:** 10.1126/sciadv.abn7063

**Published:** 2022-06-10

**Authors:** Kristian Jeppsson, Toyonori Sakata, Ryuichiro Nakato, Stefina Milanova, Katsuhiko Shirahige, Camilla Björkegren

**Affiliations:** 1 Karolinska Institutet, Department of Biosciences and Nutrition, Neo, Hälsovägen 7c, 141 83 Huddinge, Sweden.; 2 Karolinska Institutet, Department of Cell and Molecular Biology, Biomedicum, Tomtebodavägen 16, 171 77 Stockholm, Sweden.; 3 Institute for Quantitative Bioscience, Tokyo University, 1-1-1, Yayoi, Bunkyo-ku, Tokyo 113-0032, Japan.

## Abstract

Genome function depends on regulated chromosome folding, and loop extrusion by the protein complex cohesin is essential for this multilayered organization. The chromosomal positioning of cohesin is controlled by transcription, and the complex also localizes to stalled replication forks. However, the role of transcription and replication in chromosome looping remains unclear. Here, we show that reduction of chromosome-bound RNA polymerase weakens normal cohesin loop extrusion boundaries, allowing cohesin to form new long-range chromosome cis interactions. Stress response genes induced by transcription inhibition are also shown to act as new loop extrusion boundaries. Furthermore, cohesin loop extrusion during early S phase is jointly controlled by transcription and replication units. Together, the results reveal that replication and transcription machineries are chromosome-folding regulators that block the progression of loop-extruding cohesin, opening for new perspectives on cohesin’s roles in genome function and stability.

## Teaser

Spatial genome organization is controlled by transcription and stalled replication machineries that limit cohesin loop extrusion.

## INTRODUCTION

Cohesin belongs to the family of structural maintenance of chromosome (SMC) protein complexes and was initially identified as a tether of sister chromatids, the products of chromosome replication (*1*, *2*). This cohesion, established in close vicinity to the advancing replication fork and removed at anaphase onset, is essential for correct chromosome alignment and segregation during mitosis (*3*–*5*). Cohesin complexes involved in sister chromatid cohesion are stably associated to chromosomes (*6*), and do not dissociate from chromosomes when the cohesin loading factor Scc2 [human Nipped-B-like (NIPBL)] is inhibited (*7*). In addition to being localized at centromeres, these stable complexes are found in between convergently oriented genes in *Saccharomyces cerevisiae* (*8*). A correlation between cohesin localization and convergently oriented gene pairs has also been detected in human cells (*9*). This positioning is generally thought to reflect how transcribing RNA polymerases push cohesin into place, but direct evidence for this is lacking. Later investigations using genome-wide chromosome conformation capture (Hi-C) techniques have revealed that cohesin also creates chromosome loops (*10*, *11*). Work in mammalian and insect cells show that interphase chromosomes are organized into so-called topologically associated domains (TADs), which are large chromosomal regions within which chromosome cis interactions occur more frequently than with neighboring regions (*12*–*14*). Current data indicate that cis interactions within a TAD can be largely explained by dynamic loop extrusion by cohesin (*11*, *15*) and that the insulator protein CTCF (CCCTC-binding factor) plays an essential role in TAD boundary formation in mammalian cells (*12*, *16*). More recent analyses show that *S. cerevisiae* sister chromatids are shaped into loops and domains by cohesin (*17*–*19*). These structures are considerably smaller than mammalian TADs, and their boundaries are found at the previously identified cohesin binding sites between convergently transcribed genes and at centromeres (*17*–*22*). Thus, in addition to stably associated cohesin complexes that mediate cohesion, the replicated *S. cerevisiae* genome appears to be organized by a subfraction of loop-extruding, dynamic cohesin complexes. The loop-extruding cohesin complexes are continuously unloaded by Wpl1 [human wings apart-like (WAPL), also know as Rad61] (*17*) but occasionally reach the boundaries of a domain, thereby linking two cohesin binding sites in a loop. Cohesin-dependent loops and domains with boundaries between convergently oriented genes can also be induced on unreplicated *S. cerevisiae* chromosomes (*17*, *18*), which shows that the boundaries for loop extrusion are independent of the stably associated cohesin complexes that mediate sister chromatid cohesion. This opens up the possibility that the boundaries are created by, or as a consequence of, transcription.

## RESULTS

**Fig. 1.**
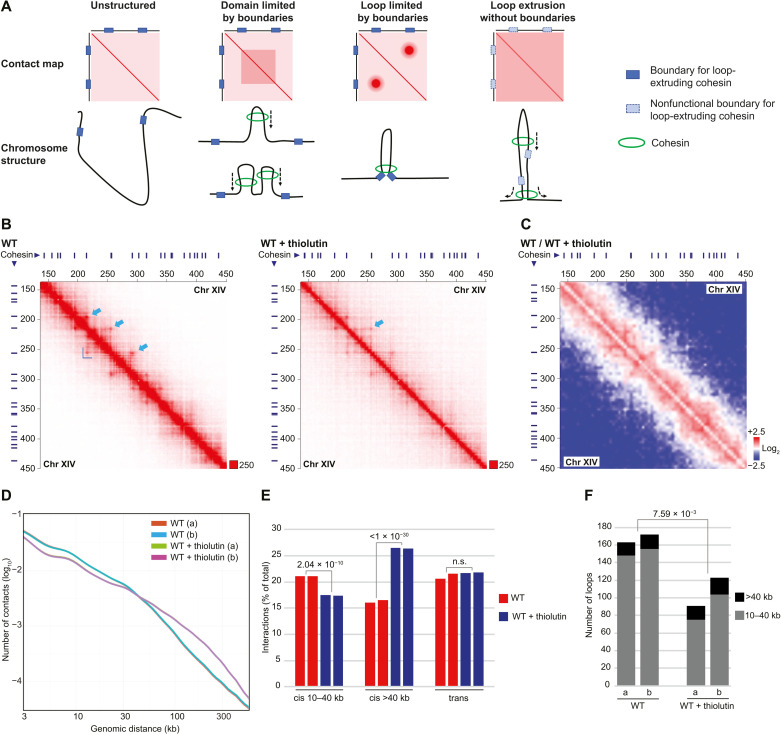
Transcription inhibition removes cohesin loop extrusion barriers, triggering the formation of novel long-range cis interactions. (**A**) Schematic illustration of cohesin loop extrusion and corresponding Hi-C contact maps in the presence of functional, or nonfunctional, loop extrusion boundaries. (**B**) Normalized Hi-C contact maps (2-kb binning) showing cis interactions along the arm of chromosome (Chr) XIV (150 to 450 kb from left telomere) in *G2/M*-arrested, untreated, and thiolutin-treated WT cells. Blue lines on top and to the left of the panels, cohesin binding sites; dark blue L shape within the panels, example of a domain; light blue arrows, examples of loop anchors. (**C**) Normalized Hi-C ratio maps (without binning) comparing chromosome cis interactions in untreated and thiolutin-treated *G2/M*-arrested WT cells along the same chromosomal regions as depicted in (B). (**D**) Contact probability plots as function of genomic distance comparing interactions in *G2/M*-arrested, untreated, and thiolutin-treated WT cells. (**E**) Quantification of cis and trans interactions in *G2/M*-arrested, untreated, and thiolutin-treated WT cells. (**F**) Number of loops anchored at cohesin sites in *G2/M*-arrested, untreated, and thiolutin-treated WT cells. (E) and (F) display results from two biological repeats, and statistical significance is indicated with *P* values from binominal tests. n.s., not significant.

**Fig. 2.**
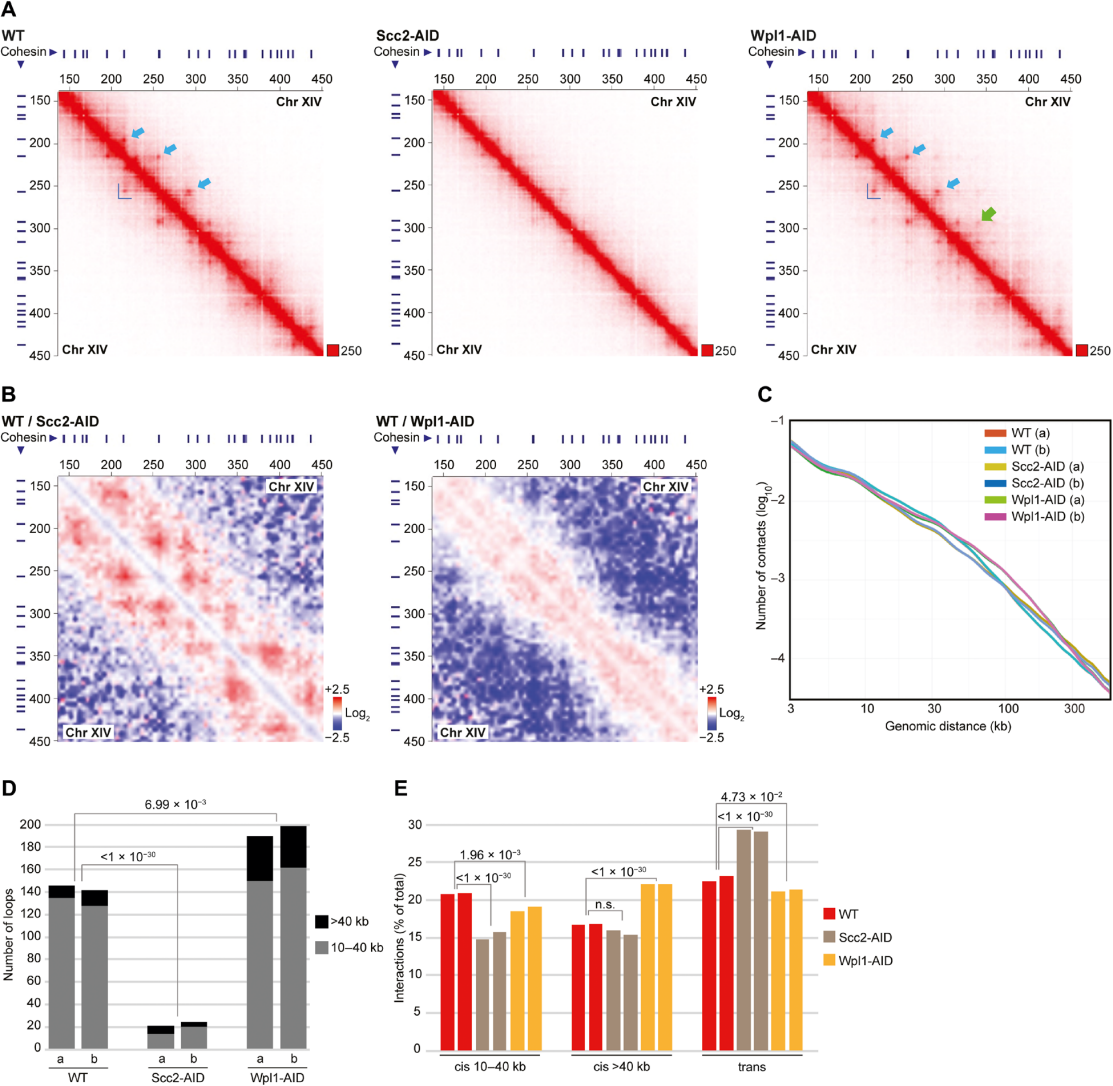
Depletion of the cohesin loader Scc2 in *G2/M* phase disrupts chromosome loop formation. (**A**) Normalized Hi-C contact maps (2-kb binning) showing cis interactions along the arm of chromosome XIV (150 to 450 kb from left telomere) in *G2/M*-arrested WT cells or in cells after depletion of Scc2 and Wpl1 (Scc2-AID, Wpl1-AID) in *G2/M* arrest. Highlights as in Fig. 1B), with an additional light green arrow showing a loop anchor only detected after Wpl1 depletion. (**B**) Normalized Hi-C ratio maps (without binning) comparing chromosome cis interactions in *G2/M*-arrested WT cells with those detected in cells depleted of Scc2 and Wpl1 (Scc2-AID, Wpl1-AID), along the same chromosomal regions as depicted in (A). (**C**) Contact probability plots as function of genomic distance displaying interactions in *G2/M*-arrested WT cells, or after depletion of Scc2 and Wpl1 (Scc2-AID, Wpl1-AID) in *G2/M* arrest. (**D**) Number of loops anchored at cohesin sites in *G2/M*-arrested WT cells, or after depletion of Scc2 and Wpl1 (Scc2-AID, Wpl1-AID) in *G2/M* arrest. (**E**) Quantification of cis and trans interactions in *G2/M*-arrested WT cells, or after depletion of Scc2 and Wpl1 (Scc2-AID, Wpl1-AID) in *G2/M* arrest. (D) and (E) display results from two biological repeats, and statistical significance is indicated with *P* values from binominal tests.

To test whether transcription creates boundaries for loop-extruding cohesin (Fig. 1A), we performed genome-wide Hi-C analysis to determine how thiolutin, an inhibitor of yeast RNA polymerases (*23*), influences chromosome structure in *G2/M*-arrested *S. cerevisiae* cells. Chromatin immunoprecipitation sequencing (ChIP-seq) and quantitative. ChIP quantitative polymerase chain reaction (ChIP-qPCR) analysis of the RNA polymerase II (RNA pol II) subunit Rpo21 showed that the level of RNA pol II is reduced in 93.5% of all open reading frames (ORFs) 30 min after addition of the drug to *G2/M*-arrested cells (figs. S1, A to C, and S2A). Consequently, thiolutin triggers RNA pol II depletion in a vast majority of the convergently oriented gene pairs that flank cohesin binding sites. Thiolutin-dependent inhibition of transcription was further confirmed by spike-in total RNA sequencing (RNA-seq), which showed a clear reduction of nascent RNA (fig. S1D). Having established that transcription inhibition by thiolutin leads to a reduction of chromosome-bound RNA pol II, we performed Hi-C experiments under the same conditions. Contact and ratio maps from experiments on wild-type (WT) cells indicated that thiolutin treatment indeed weakens interactions between cohesin binding sites (identified by ChIP-seq; see Materials and Methods) and triggers a simultaneous appearance of new, long-ranging chromosome cis interactions (Fig. 1, B and C, and figs. S2A and S3A). We confirmed the thiolutin-induced transition from short- to long-range cis interactions by contact probability analysis and by quantification of chromosome interactions from two independent experiments of each condition (Fig. 1, D and E). In line with weakened interactions between cohesin sites, the number of loops and domains flanked by cohesin sites significantly decrease upon thiolutin treatment. (Fig. 1F and fig. S3B). The new long-range cis interactions appearing after transcription inhibition are randomly located as no increase in long loops or large domains was observed after thiolutin treatment. Loops, domains, and short-range cis interactions were also significantly reduced after depletion of Scc2 in *G2/M*-arrested cells (Fig. 2, A to E, and figs. S2, B and F, and S3, A and C), thereby confirming that these structures depend on continuous loading of cohesin. Aggregate peak analysis (APA) of cis interactions between cohesin binding sites at increasing chromosome distances also revealed a substantial, although not complete, reduction in interactions (fig. S3D). In contrast to the thiolutin-treated cells, no new long-range cis interactions were observed after depletion of Scc2, which instead triggered an increase in chromosome trans interactions (Fig. 2E). Long-range cis interactions have, however, been shown to increase in cell lacking the cohesin unloader Wpl1, in which loop-extruding cohesin complexes remain longer on chromosomes than in WT cells (*17*). To be able to compare this alteration to that appearing after thiolutin treatment, we performed Hi-C analysis after *G2/M*-specific depletion of Wpl1 (fig. S2, B and F). This confirmed an increase in long-range cis interactions (Fig. 2, A to C and E, and fig. S3, A and D) but, in contrast to thiolutin-treated cells, also revealed a significant increase in the number of loops and domains in the Wpl1-depleted cells (Fig. 2D and fig. S3C). Contact probability plots also showed that Wpl1 depletion only affects interactions ranging between ≈40 to ≈300 kb, that is, inducing a more limited change as compared to thiolutin treatment in which interactions up to, and above, 500 kb were detected (Figs. 1D and 2C). Collectively, the Hi-C analysis shows that transcription inhibition induces changes in chromosomes conformation that is different from the effects caused by prevention of cohesin loading or unloading. In further support of this, aggregated plots of all *S. cerevisiae* pericentromeric regions reveal that the barrier function of centromeres is maintained in thiolutin-treated and Wpl1-depleted cells but weakened after Scc2 depletion (fig. S3, E and F) (*21*). Quantitative analysis of pericentromere-anchored cis interactions provided additional evidence for an increase in long-range cis interactions after thiolutin treatment and Wpl1 depletion and a reduction in Scc2-depleted cells (fig. S3, G and H). We also performed Hi-C analysis after *G2/M*-specific inactivation of RNA pol II using the temperature-sensitive allele *rpb1-1* for 120 min, known to reduce the level of chromosome-bound RNA pol II (*24*). Similar to the effect of thiolutin, this caused a reduction in loops, domains, and short-range cis interactions combined with concomitant increase in long-range cis interactions and maintenance of centromere barrier function (fig. S4, A to G). However, in contrast to the effect detected in thiolutin-treated cells, trans interactions increase in *rpb1-1* cells, and pericentromere-anchored cis interactions remained unchanged (fig. S4, F and H). This is likely due to the extended period of inhibition needed to deplete RNA pol II from chromosomes in the mutant, which increases the risk of also reducing chromosome-bound cohesin, reported to be loaded at promoters of actively transcribed genes (*8*). Together, the results indicate that transcription inhibition associated with depletion of chromosome-bound RNA pol II removes chromosome barriers for loop-extruding cohesin complexes. This allows the complex to translocate beyond normal loop and domain boundaries, thereby reducing their numbers, and to create more long-range, randomly located, cis interactions (Fig. 1A). To challenge this idea, we performed Hi-C analysis on cells in which Scc2 had been depleted before addition of thiolutin. This showed that inhibition of cohesin loading significantly reduced the number of thiolutin-induced long-range cis interactions, supporting the idea that they are a result of cohesin-dependent loop expansion (fig. S4, I and J). The remaining thiolutin-induced long-range cis interactions in Scc2-depleted cells are likely due to a certain level of remaining chromosome-associated, loop-forming cohesin complexes (fig. S3D) and/or cohesin-independent mechanisms.

**Fig. 3.**
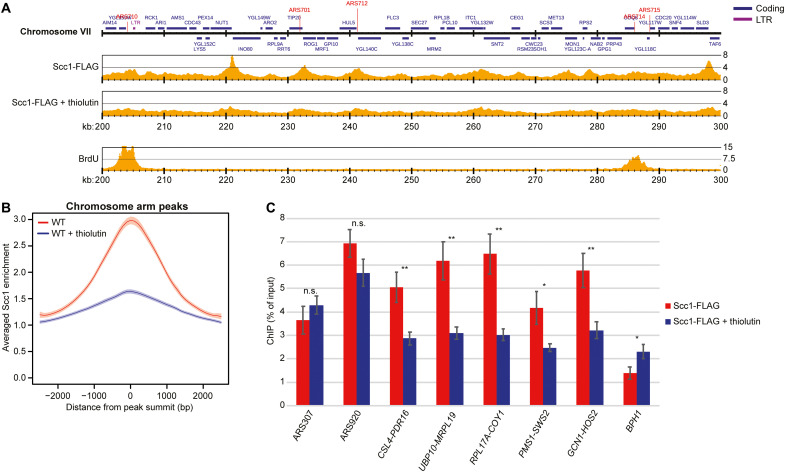
Transcription inhibition delocalizes dynamic cohesin from its normal binding sites between convergently oriented gene pairs. (**A**) Scc1-FLAG enrichment on chromosome VII (200 to 300 kb from left telomere) based on normalized ChIP-seq analysis of untreated and thiolutin-treated WT cells arrested in S phase by HU (two upper maps). The lowest map shows 5-bromo-2′-deoxyuridine (BrdU) incorporation in the same chromosomal region in synchronized WT cells arrested in S phase by HU (*23*). Note that replication forks have progressed a few kilobase pairs from the early firing origins ARS710 and ARS714, leaving most of the region in view unreplicated. The *y* axis shows fold enrichment of ChIP / input in linear scale, and the *x* axis shows chromosomal positions. Blue and purple horizontal bars in the uppermost genomic region panel denote coding regions and long terminal repeats (LTRs), respectively, and red vertical lines indicate replication origins [autonomously replicating sequence (ARS)]. (**B**) Averaged Scc1-FLAG enrichment at cohesin sites along chromosome arms, based on the analysis presented in (A). Shaded regions indicate the 95% confidence interval. (**C**) Chromosomal association of Scc1-FLAG at two early replication origins (ARS307 and ARS920) at selected cohesin sites (intergenic regions indicated by flanking convergently oriented gene pairs), and within the 5′ end of *BPH1* ORF, as determined by ChIP-qPCR of samples collected from cells treated as in (A). *N =* 3, n.s. *P* > 0.05, *P ≤ 0.05, **P ≤ 0.01.

If transcription inhibition allows cohesin to loop extrude beyond its normal boundaries, then the accumulation of cohesin between convergent genes is expected to diminish. In agreement with this, thiolutin has been reported to reduce cohesin accumulation at some sites along chromosome arms in *G2/M*-arrested cells (*18*). Revisiting this, we analyzed the binding pattern of the cohesin subunit Scc1 (human RAD21) by normalized ChIP-seq and ChIP-qPCR, with or without prior addition of thiolutin. This provided similar results as in (*18*), i.e., minor reduction of cohesin enrichment at some binding sites (fig. S5, A to D). Average peak plots of Scc1 enrichment at cohesin binding sites along chromosome arms revealed a general broadening of peaks (fig. S5B), potentially reflecting a thiolutin-induced splitting of Scc1 peaks into two parts (fig. S5A), and indicating a change in positioning in response to transcription inhibition. The limited change is likely due to remaining, stably associated, cohesive cohesin complexes, which also have been observed after Scc2 inhibition in *G2/M*-arrested cells (*25*, *26*). To enable analysis of dynamic cohesin complexes alone, we investigated the effect of thiolutin on the chromosomal association of Scc1 in cells arrested in early S phase by addition of hydroxyurea (HU), in which the short, 5- to 10-kb, replicated regions have been mapped with high precision [see for example (*27*)]. In the unreplicated regions, which are devoid of cohesive cohesin complexes, thiolutin triggered a general and significant reduction of Scc1 enrichment in intergenic regions between convergently expressed genes (Fig. 3, A to C, and fig. S2C). In contrast, cohesin association to the stalled forks was unaltered by thiolutin, and the complex was enriched at sites distant to its normal binding sites, such as within the ORFs of genes >3 kb [Fig. 3C (*BPH1*) and fig. S5E; (*28*)]. These results support the Hi-C analysis, which indicates that transcription inhibition removes barriers that block the progression of dynamic loop-extruding cohesin (Figs. 1 and 2, and figs. S3 and S4), thereby reducing cohesin accumulation between convergent genes, while leaving the accumulation at stalled forks unaltered and increasing enrichment in regions of chromosomes that normally display low levels of cohesin association.

**Fig. 4.**
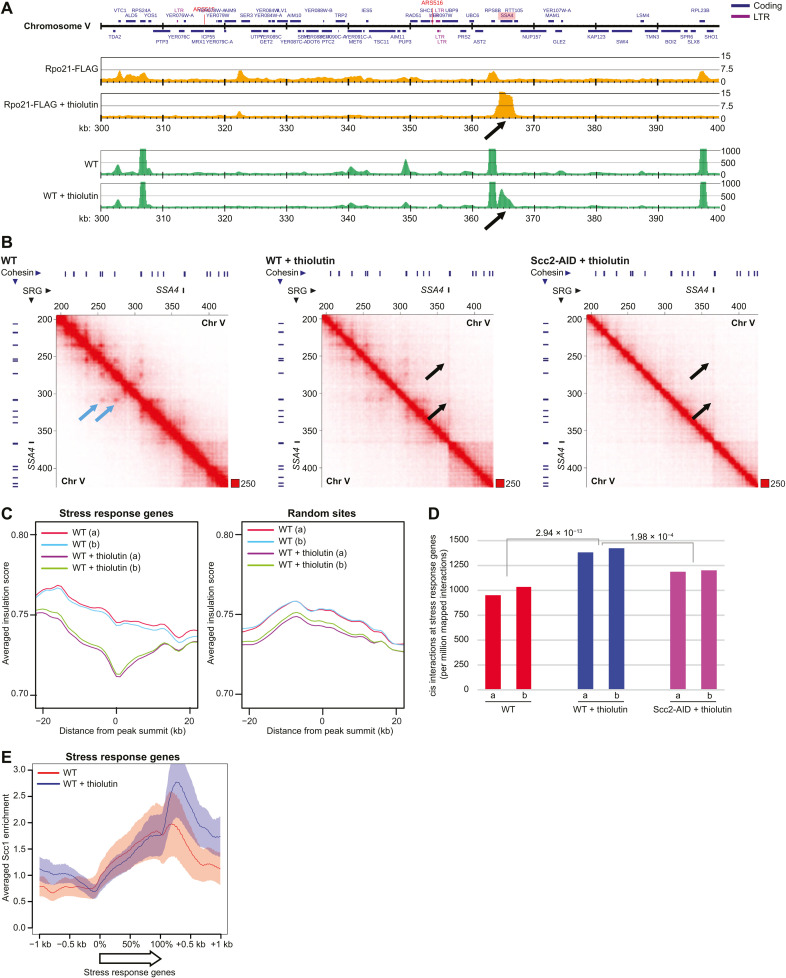
Highly expressed genes create novel chromosome loop boundaries. (**A**) Rpo21-FLAG enrichment on chromosome V (300 to 400 kb from left telomere) (upper two maps) and RNA levels based on spike-in normalized total RNA-seq, with the *y* axis denoting spike-in normalized read numbers (lower two maps), in the absence or presence of thiolutin in *G2/M*-arrested WT cells. Black arrows indicate thiolutin-induced Rpo21-FLAG accumulation at, and up-regulation of, the stress response gene *SSA4*. (**B**) Normalized Hi-C contact maps (2-kb binning) showing cis interactions along the arm of chromosome V (200 to 420 kb from left telomere) in *G2/M*-arrested, untreated, and thiolutin-treated WT and Scc2-depleted cells (Scc2-AID). Black arrows indicate boundary formation and cis interactions at the *SSA4* gene. Light blue arrows highlight loop anchors that are weakened by thiolutin. Blue and black lines on top and to the left of the panels, cohesin binding sites and stress response gene (SRG), respectively. (**C**) Averaged insulation score plots based on normalized Hi-C data from *G2/M*-arrested, untreated, and thiolutin-treated WT cells. The analysis focuses on 40-kb regions spanning 38 up-regulated stress response genes that display thiolutin-induced increase in Rpo21-FLAG association (left) or 38 random sites (right). (**D**) Quantification of cis interactions (>10 kb) anchored at stress response genes in *G2/M*-arrested, untreated, and thiolutin-treated WT or Scc2-depleted cells. Statistical significance is indicated by *P* values from a binominal test. (C) and (D) display results from two biological repeats. (**E**) Averaged Scc1-FLAG enrichment at stress response genes, based on ChIP-seq analysis presented in fig. S5A.

In addition to functioning as a transcriptional inhibitor, thiolutin has been reported to induce a cellular stress response (*29*). In line with this, a small number of genes display increased RNA pol II enrichment after thiolutin addition (examples in Fig. 4A and fig. S6, A and E). Similarly, RNA-seq detected a small number of up-regulated genes (fig. S6, B, D, and E). This provided an additional tool to test whether transcription controls cohesin-dependent loop formation. On the basis of results from normalized ChIP-seq of Rpo21 and spike-in total RNA-seq in thiolutin-treated cells, we identified 38 highly up-regulated thiolutin-induced stress response genes that were used in subsequent analysis of chromosome organization (fig. S6C). Hi-C contact maps indicated that the stress response genes created new loop extrusion boundaries (Fig. 4, A and B, and fig. S6F), which was confirmed by insulation score analysis and quantification of cis interactions anchored at the induced genes (Fig. 4, C and D). These interactions were significantly reduced by depletion of Scc2 before the addition of the drug, confirming that they depend on cohesin loading (Fig. 4, B and D). This was further supported by the observation that thiolutin causes cohesin to accumulate at the 3′ end of the stress response genes, which also indicates that RNA pol II limits loop expansion via head-on collisions with cohesin complexes (Fig. 4E). Together, this provides additional evidence that the transcription machinery inhibits the progression of loop-extruding cohesin, thereby controlling chromosome loop organization.

**Fig. 5.**
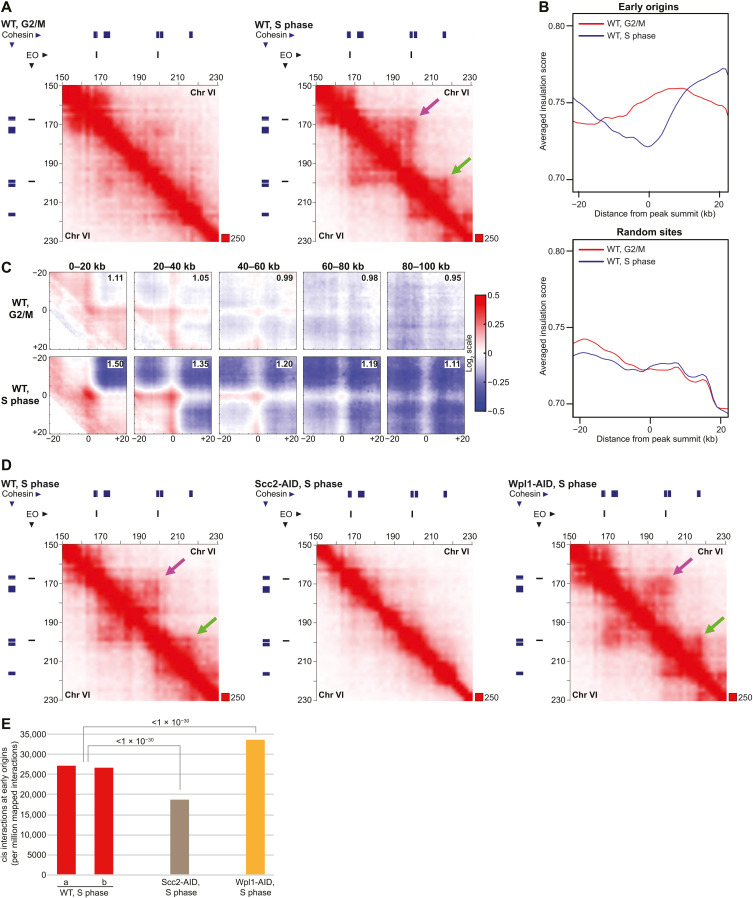
Stalled replication forks act as barriers for loop extruding cohesin. (**A**) Normalized Hi-C contact maps (2-kb binning) showing cis interactions along the arm of chromosome VI (150 to 230 kb from left telomere) in *G2/M*-arrested WT cells and S phase–arrested WT cells. Purple arrow, interactions between neighboring early replication origins; green arrow, replication origin-cohesin site interaction formed in S phase. Blue and black lines on top and to the left of the panels, cohesin binding sites and early firing origin (EO), respectively. (**B**) Averaged insulation score plots based on normalized Hi-C data as in (A). The analysis focuses on 40-kb regions spanning early replication origins on chromosome arms (top) or random sites (bottom). (**C**) APA centered on interactions between pairs of early origins and cohesin sites, separated by increasing chromosomal distances as indicated on top of the panels. The analysis is based on normalized Hi-C data from *G2/M*-arrested, or S phase–arrested WT cells as in (A). The strength of the central loop signal is displayed in the upper right corner of each panel. (**D**) Normalized Hi-C contact maps in S phase–arrested WT cells, or in S phase–arrested cells predepleted of Scc2 and Wpl1 (Scc2-AID, Wpl1-AID), as in (A). (**E**) Quantification of cis interactions (>10 kb) anchored at early replication origins in S phase–arrested WT cells or in S phase–arrested cells predepleted of Scc2 and Wpl1 (Scc2-AID, Wpl1-AID). Statistical significance is indicated with *P* values from a binominal test. Results from two biological repeats are shown for WT.

The observation that transcription inhibition reduces cohesin chromosomal association in unreplicated regions in HU-arrested cells (Fig. 3, A to C) opens for that these dynamic cohesin complexes also form loops under these conditions. Hi-C analysis of HU-arrested WT, Scc2-, and Wpl1-depleted cells showed that this is indeed the case. In addition to revealing loops anchored at cohesin binding sites between convergently oriented gene pairs, the resulting contact maps disclosed strong loop extrusion boundaries at stalled replication forks (Fig. 5A and fig. S7A). This indicates that loop-extruding cohesin is blocked by the stalled replication machinery, which was supported by insulation score analysis and APA focusing on interactions between pairs of cohesin binding sites and early replication origins along chromosome arms (Fig. 5, B and C). Contact maps also indicated that interactions between stalled forks and cohesin sites were weakened and strengthened by Scc2 and Wpl1 depletion, respectively, which was confirmed by quantification of cis interactions anchored at origins (Fig. 5, D and E, and fig. S7B). Together, this shows that stalled replication forks limit cohesin-dependent loop extrusion in HU-arrested cells, indicating that replication and transcription machineries jointly control chromosome looping during normal S-phase progression.

## DISCUSSION

**Fig. 6.**
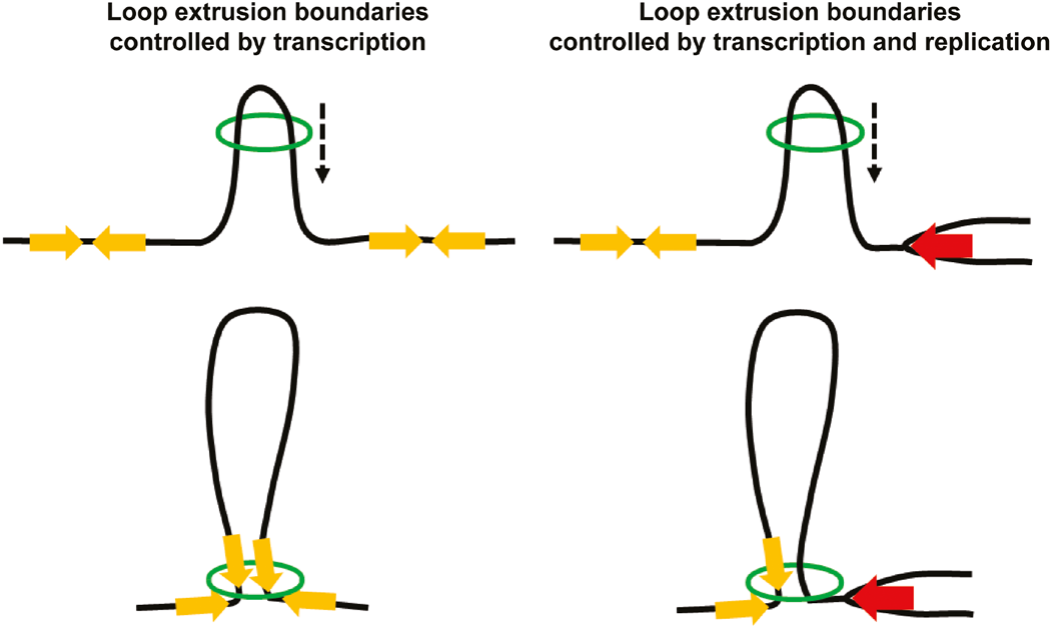
Schematic model of how transcription and replication control cohesin loop extrusion boundaries. Loop-extruding cohesin complexes (green open circles) are blocked by head-on collisions with transcribing RNA polymerases in convergently oriented genes (yellow arrows), which thereby constitute loop extrusion boundaries. Stalled replication forks (red arrows) also create chromosome interaction boundaries by blocking loop-extruding cohesin complexes. The resulting positioning of cohesin complexes in the vicinity of the polymerases might underlie cohesin’s functions in transcription regulation and fork stability. Black dashed arrows indicate the direction of cohesin movement along chromosomes during extrusion.

Together, the presented results reveal an unforeseen interplay between transcription, replication, chromosome three-dimensional organization, and cohesin functions (Fig. 6A). Cohesin’s role in chromosome looping (*10*, *11*, *15*) and transcription-dependent localization of the complex in both yeast and human cells are well established (*8*, *9*, *30*). However, the relationship between these two features has previously not been directly addressed, and the current model, originating from analyses of stably bound, cohesive complexes, proposes that transcription pushes cohesin along chromosomes (*8*). Even if this might still be true for complexes involved in sister chromatid cohesion, our analysis indicates that transcription-dependent positioning of dynamic cohesin complexes reflects a barrier function for transcription. The results also imply that the positioning of cohesin at 3′ ends of convergently oriented genes is caused by head-on collisions between the convergently oriented transcription units and cohesin complexes moving into the intergenic region from opposite sides (Fig. 6A). Moreover, our investigation opens for the possibility that stably bound, cohesive complexes in between convergently oriented genes are pushed in place by loop-extruding cohesins and not the transcription machinery itself. The observation that bacterial SMC complexes that translocate along newly replicated chromosomes are blocked by convergently oriented genes (*31*, *32*) indicates that the here-observed cohesin-transcription interplay reflects an evolutionary conserved feature of SMC complexes. The evolutionary conservation of transcription as a roadblock for loop-extruding cohesin is further supported by investigations showing that TAD boundaries correlate with active transcription in many species [reviewed in (*33*)]. This said, the overall effect of transcription on chromosome loop formation is expected to vary depending on the presence of other barriers, such as the CTCF insulator protein (*12*, *13*, *34*). The recently published results showing that cohesin is required for increased tethering of CTCF sites in response to transcription inhibition strengthen the notion that transcription can act in parallel with CTCF-dependent loop control (*35*).

In addition to revealing a function for transcription in loop formation, our results also open up the possibility that cohesin’s role in gene regulation might be executed not only by chromosome loop organization but also via a direct association with the transcription machinery. The observation that stalled replication forks are barriers for loop-extruding cohesin complexes also has several implications. First, the results are consistent with the possibility that the previously reported accumulation of cohesin at HU-arrested replication forks reflects how cohesin complexes arrive at the stalled fork by loop extrusion instead of being recruited from a soluble fraction (Fig. 6A) (*28*). Such a mechanism also opens for new models for S phase–specific functions of cohesin, including establishment of sister chromatid cohesion, which could depend on loop-extruding cohesins that are converted to cohesive complexes behind the fork. Second, cohesin might function not only on newly formed chromatids but also in front of the fork, where the loop-extruding activity could influence fork progression and stability (*28*, *36*). Third, a role for the unchallenged replication machinery in determining chromosome looping during S phase could be an important aspect in the establishment of TADs and provides a potential mechanism for transcription-independent TAD formation during early development (*37*). Together, the revelation that both transcription and replication machineries are barriers for cohesin-dependent loop extrusion sets the stage for future analysis of chromosome organization and cohesin function from new perspectives.

## MATERIALS AND METHODS

### Yeast strains, growth conditions, protein degradation, and transcription inhibition

All *S. cerevisiae* strains are of W303 origin with the modifications listed in table S1. Cells were cultured in YEP medium [1% yeast extract, 2% peptone, and adenine (40 μg ml^−1^)] supplemented with 2% glucose (YEPD). For arrest in *G2/M*, benomyl (Sigma-Aldrich, 381586)–containing YEPD medium was added to cells growing logarithmically to a final concentration of 80 μg ml^−1^. Cell cultures were then incubated for 90 min at 30°C, achieving complete *G2/M* arrest. For synchronization in S phase, α-factor mating pheromone (3 μg ml^−1^; custom peptide WHWLQLKPGQPMY, Sigma-Aldrich) was added every hour (a total of three additions) to cells growing logarithmically at 25°C. Upon complete G1 arrest, cells were released into medium containing 0.2 M HU (Sigma-Aldrich, H8627), and S phase was allowed to progress at 25°C. For transcription shutoff and degradation of Scc2 and Wpl1 in *G2/M* arrest, auxin (3-indoleacetic acid, Sigma-Aldrich, I2886) and doxycycline (Sigma-Aldrich, D9891) were added for 1 hour at the final concentration of 1 mM and 5 μg ml^−1^, respectively. For transcription shutoff and degradation of Scc2 and Wpl1 in a synchronized S phase, auxin and doxycycline were first added to G1-arrested cells for 30 min and then for an additional hour in the HU-containing release medium at the same final concentrations as above. For transcription inhibition, thiolutin (Abcam, ab143556) was added to cell cultures for the final concentration of 20 μg ml^−1^ for 30 min. For inactivation of RNA pol II in *G2/M* arrest, temperature-sensitive *rpb1-1* cells were first arrested by benomyl for 2 hours at the permissive temperature of 25°C. Thereafter, the temperature was shifted to restrictive 37°C for 2 hours. Cell cycle progression and arrests were confirmed using standard protocol for fluorescence-activated cell sorting (FACS) analysis of ethanol-fixed, propidium iodide–stained cells (see fig. S2 for details on each experimental setup). The *Schizosaccharomyces pombe* strain (table S1) used for spike-in normalization in RNA-seq was grown in YES medium [0.5% yeast extract, 3% glucose, adenine (150 μg ml^−1^), uracil (100 μg ml^−1^), leucine (100 μg ml^−1^), lysine (100 μg ml^−1^), and histidine (100 μg ml^−1^)] at 32°C.

### Hi-C library preparation

Our Hi-C protocol was adapted for *S. cerevisiae* from (*34*). Fifty milliliters of cell culture [at an optical density (OD) of 1.0] was fixed with 3% formaldehyde for 20 min at 30°C before the reaction was quenched by adding glycine to 0.125 M final concentration for 5 min at room temperature. Cells were washed once with 1× PBS, before being resuspended in 5 ml of pre-spheroplasting buffer [100 mM Pipes (pH 9.4) and 10 mM dithiothreitol (DTT)]. The cells were incubated for 5 min at room temperature and then pelleted (1500*g* for 5 min at room temperature), before being resuspended in 5 ml of spheroplasting buffer [50 mM KH_2_PO_4_/K_2_HPO_4_ (pH 7.5), 1 M sorbitol, and 10 mM DTT]. Twenty-five microliters of 100T Zymolyase (10 mg ml^−1^; Nacalai Tesque, 07665-55) was added, and cells were incubated for 15 min at 30°C. The spheroplasts were pelleted (500*g* for 5 min at 4°C) and washed twice with ice-cold spheroplasting buffer (containing only 1 mM DTT). The spheroplasts were then resuspended in 250 μl of ice-cold Hi-C lysis buffer [10 mM tris-HCl (pH 8.0), 10 mM NaCl, and 0.2% Igepal CA630] supplemented with 50 μl 6× cOmplete protease inhibitor in water (Roche, 04693132001) and 3 μl of protease inhibitor (Sigma-Aldrich, P8215), and incubated on ice for 15 min. The spheroplasts were pelleted (1500*g* for 5 min at 4°C) and washed twice with 500 μl of ice-cold Hi-C lysis buffer. After the last wash step, the supernatant was discarded, and the spheroplasts were incubated for 6 min at 62°C. Thereafter, SDS was added to a final concentration of 0.2%, and the reaction was immediately and thoroughly mixed by inversion and incubated at 62°C for 10 min. After addition of 80 μl of H_2_O, 25 μl of 10% Triton X-100 was added to quench the SDS. The reaction was mixed by inversion and incubated at 37°C for 15 min. Thereafter, 28 μl 10× NEB DpnII buffer and 500 U of DpnII (NEB, R0543M) were added, and the chromatin was digested overnight at 37°C. At the end of the incubation, the reaction was supplemented with 250 U of DpnII and incubated for 1 hour at 37°C, whereafter the restriction enzyme was inactivated at 62°C for 20 min. The presence of intact and individual DNA masses throughout the spheroplasting, digestion, and ligation steps was confirmed by 4′,6-diamidino-2-phenylindole staining and microscopy. Marking and repairing DNA ends, proximity ligation, cross-link reversal, DNA shearing, size selection, biotin pulldown, preparation for Illumina sequencing, final amplification (15 cycles), and purification were performed as in (*34*). The Hi-C libraries were sequenced on Illumina HiSeq series with 150-bp paired-end sequencing according to the manufacturer’s recommendations.

### Hi-C data analysis

The Hi-C data were processed using Juicer with the default parameter set (*38*). The sequenced reads were mapped to the *S. cerevisiae* genome obtained from Saccharomyces Genome Database (SGD) (http://yeastgenome.org/). The uniquely mapped read pairs were randomly resampled and arranged in the number of the lowest sample, which was 51.2 million read pairs (see table S3). Contact matrices used for further analysis were coverage (sqrt)–normalized at 1- and 2-kb resolution with Juicer. The matrices were visualized by Juicebox (*39*). Intrachromosomal contact frequency distribution was calculated using nonduplicated valid Hi-C contact pairs at genomic distances increasing by 1 kb.

### Loop calling

Loops were identified by using the HICCUPS algorithm in Juicer with “-m 4096 -k VC_SQRT,VC_SQRT -r 500,500 -f 0.001,0.001 p 6,8 -i 12,16 -d 2500,2500” option. Briefly, loops were called by searching the enrichment of contact frequency relative to the local background with coverage (sqrt) normalization at 500-bp resolution and filtered by a false discovery rate (FDR) under 0.001 in the HICCUPS. The candidate loops were further filtered by a loop length over 10 kb, and the loops overlapping with Scc1 binding sites at both up- and downstream loop anchors were used in the subsequent analysis. Statistical significance was tested using a binomial test.

### Domain calling

Domains were identified by using the Arrowhead algorithm in Juicer with coverage (sqrt) normalization at 1- and 2-kb resolution with “-m 300 -k VC_SQRT -r 2000” and “-m 200 -k VC_SQRT -r 1000” option. The Arrowhead detects the corners of the domains to identify their boundaries. The candidate domains at 1- and 2-kb resolution were merged, and the domains overlapping with Scc1 binding sites at both up- and downstream boundaries were used in the subsequent analysis. Statistical significance was tested using a binomial test.

### Aggregated centromere plots

Aggregated intensities of pixels at centromeres were calculated with coverage (sqrt) normalization at 2-kb resolution by piling up submatrices around centromeres from the contact matrix. Each of these submatrices is a pixel square centered at a centromere.

### Calculation of cis/trans interactions

The number of uniquely mapped cis- and trans-read pairs was normalized by total read number (read pair per kilobase). For cis interactions at stress response genes, replication origins, or pericentromeric regions, uniquely mapped cis-read pairs overlapping with these regions at either or both up- and downstream sites were obtained, and the number was normalized by total read number (read pair per million mapped read pairs). Statistical significance was tested using a binomial test.

### Aggregate peak analysis

Aggregated intensities of pixels corresponding to pairs of specific sites in the contact matrices were calculated using APA with “-r 1000 -n 0 -w 20 -k VC_SQRT” option using juicertools version 1.9.9 (*34*, *38*). Briefly, APA calculates the sum of submatrices around paired genomic loci derived from the contact matrix. Each of these submatrices is a pixel square centered at a single pair of loci in the upper triangle of the contact matrix. The strength of the central loop signal was calculated by dividing the sum value of the central 11 × 11 square by the background sum value of the top-right corner square.

### Insulation score analysis

Insulation score analysis was performed as previously reported (*40*). In short, the score was calculated for every 1-kb bin as the total number of contacts formed across the bin by pairs of loci located on the either side, up to 40 kb away using coverage (sqrt)–normalized contact matrices. The score was normalized by the mean of all insulation scores.

### RNA extraction, library preparation, and RNA-seq

Forty-five milliliters of *S. cerevisiae* cell culture (at an OD of 1.0) was harvested, washed in ice-cold ribonuclease (RNase)–free water twice, and stored at −80°C. In parallel, 5 ml of logarithmically growing *S. pombe* WT cells was harvested (at an OD of 0.4) for spike-in normalization, washed in ice-cold RNase-free water twice, and stored at −80°C. A cell pellet from *S. cerevisiae* and one from *S. pombe* were combined in the same tube after thawing on ice. Thereafter, 1 ml of cold TRIzol (Thermo Fisher Scientific, 15596026) was added, and the resulting suspension filled to the brim with cold glass beads (Sigma-Aldrich, G8772). The tubes were vortexed for 8 min and set on ice before addition of 1 ml of cold TRIzol and subsequent brief vortexing. The liquid was transferred to 1.7-ml Costar tubes and centrifuged at 12000*g* for 10 min at room temperature, whereafter supernatants were transferred to new Costar tubes. Then, 200 μl of chloroform was added to the samples (Sigma-Aldrich, 1024451000), the tubes shaken by hand for 15 s, and the suspension allowed to settle before being centrifuged at 12000*g* for 15 min at 4°C. The aqueous phase was collected, and a second chloroform extraction was performed. Thereafter, 500 μl of 2-propanol (Sigma-Aldrich, 1096341000) was added to the samples, which were then inverted seven times, and allowed to precipitate for 10 min at room temperature. The precipitates were pelleted at 7500*g* for 5 min at 4°C, and the pellets were washed once with 1 ml of 70% ethanol, air-dried, and resuspended in 25 μl of RNase/DNase-free water. Twenty-five milligrams of extracted RNA was then treated with 10 U of DNase I (Sigma-Aldrich, 4716728001) for 15 min at room temperature. Large ribosomal rRNA molecules were depleted from the samples using the RiboMinus Transcriptome Isolation Kit (Thermo Fisher Scientific, K155003) according to the manufacturer’s instructions. Last, samples were prepared for sequencing using the NEBNext Ultra II Directional RNA Library Prep (NEB, E7760S) according to the manufacturer’s protocol. The RNA-seq libraries were sequenced on NextSeq 2000 (Illumina).

### RNA-seq data analysis

We used STAR version 2.7.3a to map the sequenced reads (single-end, stranded) and RSEM v1.3.1with the option “--estimate-rspd --strandedness reverse” to estimate the expression values as transcripts per kilobase million (TPM) (*41*, *42*). We further normalized TPM values using the number of RNA-seq reads mapped to transcripts from *S. pombe* using bowtie version 1.2.2 for visualization (spike-in RNA-seq) (table S4) (*43*). The scaling factors of spike-in normalization were calculated using the method of Bonhoure *et al.* (*44*). We used edgeR to obtain differentially expressed genes (DEGs, FDR < 1 × 10^−5^) based on fitted values using a generalized linear model that estimates dispersion among samples (table S5) (*45*). A volcano plot of the DEGs was generated using R, in which identified DEGs (FDR < 1× 10^−5^) were highlighted in red. To estimate the fraction of reads from nascent RNA, we mapped all reads onto the genome using Bowtie2 version 2.4.1 (*46*), and counted those mapped in the intronic regions, which are provided by Hooks *et al.* (*47*).

### ChIP, qPCR, and ChIP-seq library preparation

ChIP was performed as previously described, with the following details and modifications (*48*). One hundred milliliters of cell culture at an OD of 1.0 was cross-linked with 1% formaldehyde for 30 min at room temperature, followed by incubation at 4°C overnight. Chromatin was sheared to a size of 300 to 500 bp by sonication (Bandelin Sonopuls HD 2070.2), and IP reactions, with anti-FLAG antibody (Sigma-Aldrich, F1804) conjugated to Dynabeads Protein A (Invitrogen, 10002D), were allowed to proceed overnight at 4°C. After completing the IP and reversing cross-links as in (*48*), the DNA clean-up step was modified as follows. One hundred microliters of TE (Tris EDTA) buffer containing 10 μg of RNase A (VWR, A3832) was added to IP and input fractions, and the reactions were incubated for 1 hour at 37°C. Then, 2 μl of proteinase K (50 mg ml^−1^; Sigma-Aldrich, 000000003115879001) was added, and the reactions were incubated for 2 hours at 37°C. Last, the DNA was purified using a Qiagen PCR Purification kit according to standard instructions. ChIP-qPCR was performed using Fast SYBR Green (Applied Biosystems, 4385612) and primers listed in table S2 using an Applied Biosystem 7500 Real-Time PCR System according to the manufacturer’s instructions. The data were analyzed using two-sided *t* test, and the presented graphs show mean values from biological triplicates with error bars representing SD. For ChIP-seq, DNA from ChIP and input fractions was further sheared to an average size of approximately 150 bp by sonication (Covaris M220). Samples were then prepared for sequencing using the NEBNext Ultra II DNA Library Prep Kit for Illumina (NEB, E7645) according to the manufacturer’s protocol. The libraries were sequenced using the HiSeq 2500 platform to generate single-end 65-bp reads. Sequenced reads were mapped to the *S. cerevisiae* genome using Bowtie2 version 2.4.1 with the default parameter set (*46*). For the 5-bromo-2′-deoxyuridine (BrdU)–IP sample, we used previously published data (*27*). The reads were downloaded from Sequence Read Archive under accession IDs SRR1555037 and SRR1555038 and mapped them using Bowtie version 1.1.2 with “-n2 -k1” option because Bowtie2 does not allow color-space fastq data (see table S4 for details).

### ChIP-seq data analysis

To call peaks for Scc1, we identified bins in which the fold enrichment (ChIP / input) was more than 2.0. Peaks overlapping with long terminal repeats were excluded. To define cohesin sites on chromosome arms, Scc1 peaks that overlap with pericentromeric regions (25 kb spanning each centromere) were excluded. Stress response genes were defined as ORFs with an average Rpo21 ChIP/ input fold enrichment higher than 4.0 in the presence of thiolutin and lower than 2.0 in the dimethyl sulfoxide control condition and were classified as up-regulated in the RNA-seq analysis (FDR <1 × 10^−5^). To define replication origins that had fired under in HU arrest (i.e., early origins), we obtained a list of all origins [autonomously replicating sequences (ARSs)] in the *S. cerevisiae* genome from SGD (http://yeastgenome.org/) and selected ARSs overlapping with BrdU-IP peaks with a fold enrichment of more than 1.5. For early origins along chromosome arms, ARSs overlapping with pericentromeric regions (25 kb spanning each centromere) were excluded. To quantitatively compare the ChIP-seq peaks among samples, we normalized the ChIP-seq data using ChIP-qPCR. We calculated relative ratios of peak intensity among samples based on the four to seven qPCR sites from common peaks (table S6) and applied them as scaling factors for spike-in ChIP-seq normalization. We used DROMPAplus version 1.8. for normalizing, peak-calling, and visualizing ChIP-seq data (*49*).

### Protein extraction and Western blot

To monitor the degradation of hemagglutinin (HA)–tagged Wpl1-AID and Scc2-AID, protein extraction was performed using standard trichloroacetic acid precipitation or as in (*41*), respectively. Western blot membranes were detected using anti-HA antibody, clone 12CA5 (Roche, 1666606).

## Notes

View/request a protocol for this paper from *Bio-protocol*.
