# Supplementary material for: Cohesin-dependent chromosome loop extrusion is limited by transcription and stalled replication forks
Source: Sci Adv. 2022 Jun 10;8(23):eabn7063. doi: 10.1126/sciadv.abn7063 (PMC9187231; doi:10.1126/sciadv.abn7063)
Supplement: Supplementary file 1 — Figs. S1 to S7 Tables S1 and S2 [file sciadv.abn7063_sm.pdf]

Science Advances
AAAS

Supplementary Materials for

Cohesin-dependent chromosome loop extrusion is limited by transcription and stalled replication forks

Kristian Jeppsson *et al.*

Corresponding author: Kristian Jeppsson, kristian.jeppsson@ki.se; Camilla Björkegren, camilla.bjorkegren@ki.se

Sci. Adv. **8**, eabn7063 (2022)
DOI: 10.1126/sciadv.abn7063

The PDF file includes:

Figs. S1 to S7
Tables S1 and S2
Legends for tables S3 to S6

Other Supplementary Material for this manuscript includes the following:

Tables S3 to S6

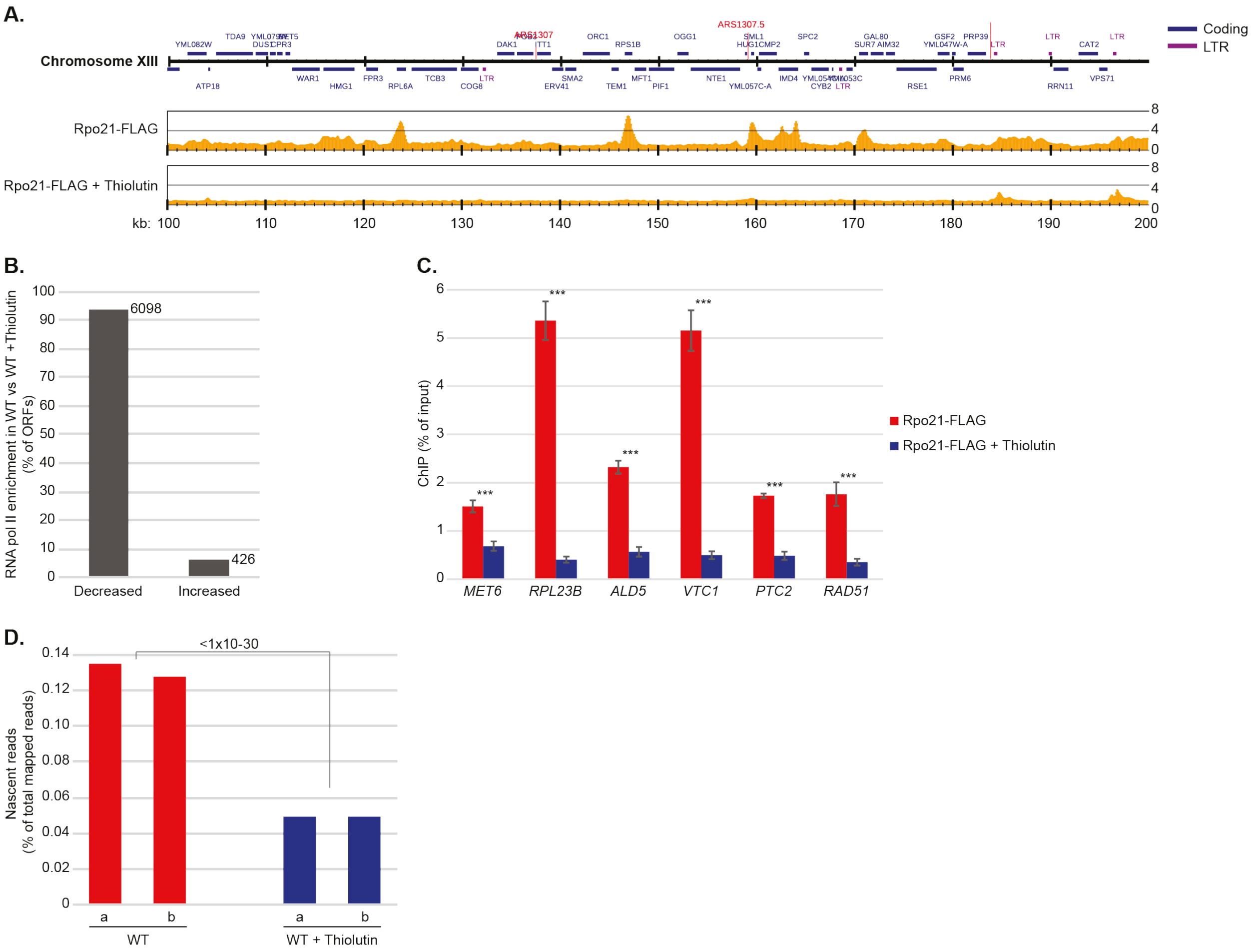

Fig. S1. Thiolutin treatment reduces RNA pol II association in most open reading frames, and reduces nascent RNA levels. A. Rpo21-FLAG enrichment on chromosome XIII (100-200 kb from the left telomere) determined by normalized ChIP-seq in the absence and presence of thiolutin in G2/M-arrested cells. The Y-axis shows fold enrichment of ChIP / input in linear scale, the X-axis shows chromosomal positions. Blue and purple horizontal bars in the genomic region panel denote coding regions and long terminal repeats (LTRs), respectively, red horizontal lines indicate replication origins (ARS). **B**. Percentage of *S. cerevisiae* open reading frames (ORFs) with higher or lower level of RNA pol II enrichment after treatment with thiolutin. The result is based on normalized ChIP-seq analysis of Rpo21-FLAG in G2/M-arrested cells as in A). Rpo21-FLAG enrichment levels were reduced in 93.5% of ORFs, increased in 6.5 % of the 6524 ORFs. **C.** Chromosome association of Rpo21-FLAG in indicated ORFs as revealed by ChIP-qPCR. N=3, $p \leq 0.001$ for all sites. **D.** Nascent RNA reads determined by spike-in total RNA-seq in G2/M-arrested, untreated and thiolutin-treated wild type (WT) cells as in A). Results from two biological repeats are shown.

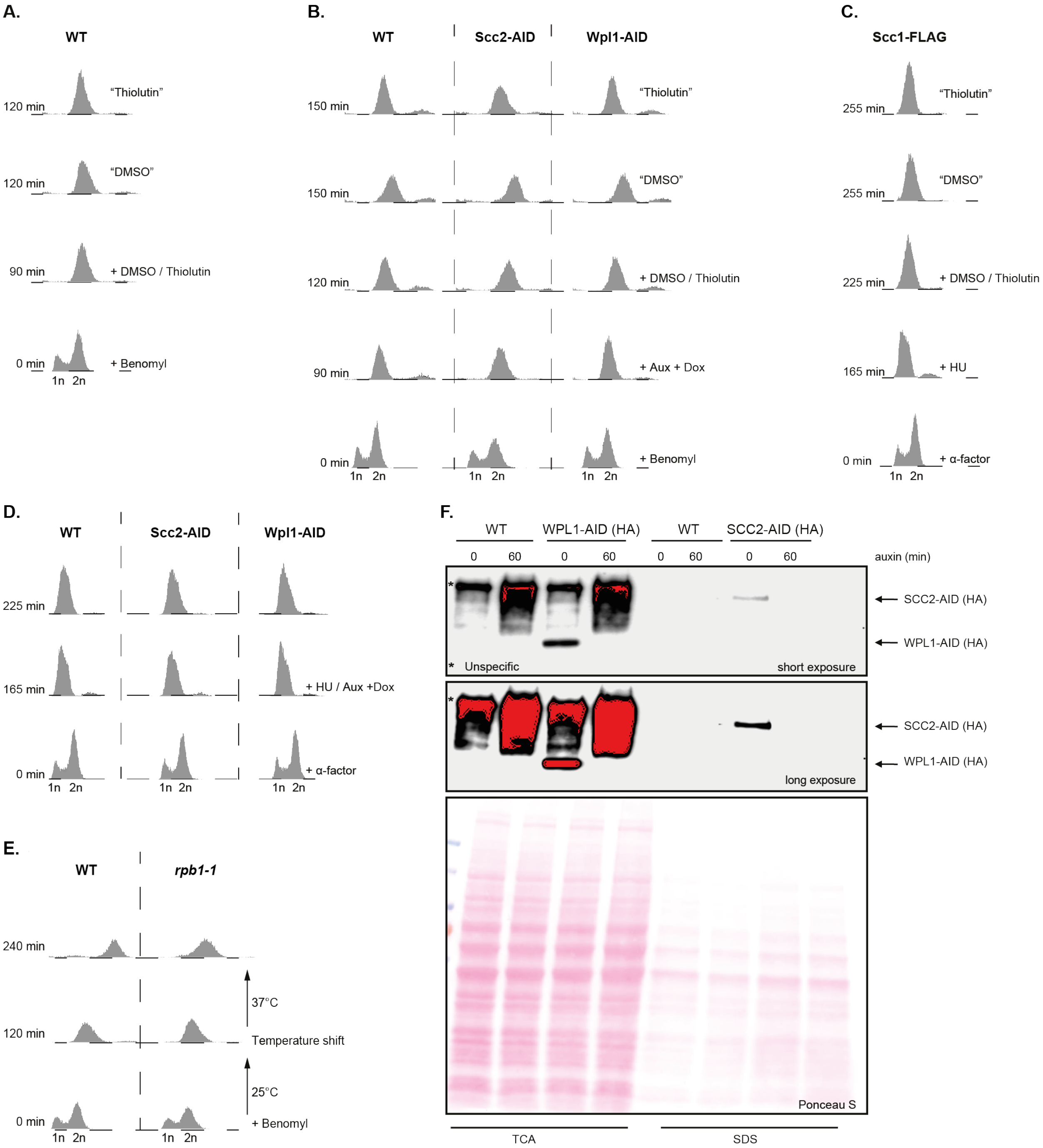

Fig. S2. Representative analyses of cell cycle progression and Scc2 and Wpl1 depletion. A. Fluorescence-activated cell sorting (FACS) analysis of logarithmically growing wild type (WT) cells initially arrested in G2/M by 90 minutes benomyl treatment. Thereafter, the cultures were split in two and treated with either DMSO or thiolutin for 30 minutes under maintained G2/M-arrest, when samples for Hi-C, ChIP-seq or RNA-seq analysis were collected. Experiments presented in Figs. 1B-F, 4A, C and E, 5A-C (G2 samples), S1A-D, S3A-B, E and G, S5A-D, S6A-F, S7A (left panel) were performed using this growth protocol. **B**. FACS analysis of indicated cell types, samples for Hi-C, ChIP-seq and ChIP-qPCR were collected at the 150 minutes time point.

Logarithmically growing cells were first arrested in G2/M by 90 minutes benomyl treatment, and subsequently treated with auxin and doxycycline for 30 minutes to deplete Scc2 or Wpl1. Thereafter, the cultures were split into two and treated with either DMSO or thiolutin for 30 minutes under maintained G2/M-arrest and Scc2- and Wpl1-depleting conditions. Experiments presented in Figs. 2A-E, 4B and D, S3C-D, F and H, S4I and J, were performed using this growth protocol. **C**. FACS analysis of Scc1-FLAG cells first arrested in G1 by addition of the alpha-factor pheromone for 165 minutes, and subsequently released into medium containing hydroxyurea to arrest cells in early S-phase. After 60 minutes, half of the culture was treated with either DMSO or thiolutin for 30 minutes when samples were collected. Experiments presented in Figs. 3A-C and S5E were performed using this growth protocol. **D**. FACS analysis of indicated cell types, first arrested in G1 by addition of the alpha-factor pheromone for 165 minutes. Thereafter, the cells were released into medium containing hydroxyurea to arrest cells in early S-phase (WT, Scc2- and Wpl1-AID), and auxin and doxycyline to deplete Scc2 or Wpl1 (Scc2-AID, Wpl1-AID). Samples for Hi-C analysis, ChIP-seq and ChIP-qPCR were collected after an additional 60 minutes. Results from S-phase arrested cells presented in Figs. 5A-E and S7A-B were obtained using this growth protocol. **E**. FACS analysis of indicated cell types, samples for Hi-C were collected at the 240 minutes time point. Logarithmically growing cells were first arrested in G2/M by 120 minutes benomyl treatment at 25°C, before cultures were shifted to 37°C for 120 minutes to inactivate the temperature-sensitive RNA pol II allele, *rpb1-1*. Experiments presented in Fig. S4A-H were performed using this growth protocol. **F**. Representative Western blots showing auxin- and doxycycline-induced degradation of Scc2- and Wpl1-AID with a HA-epitope integrated at their N-terminus. Note that the samples for detection of Wpl1 and Scc2 were prepared using different protein extraction methods using trichloroacetic acid (TCA) or sodium dodecyl sulfate (SDS). Bottom panels show PonceauS-stained transfer-membranes demonstrating equal loading within each set of samples.

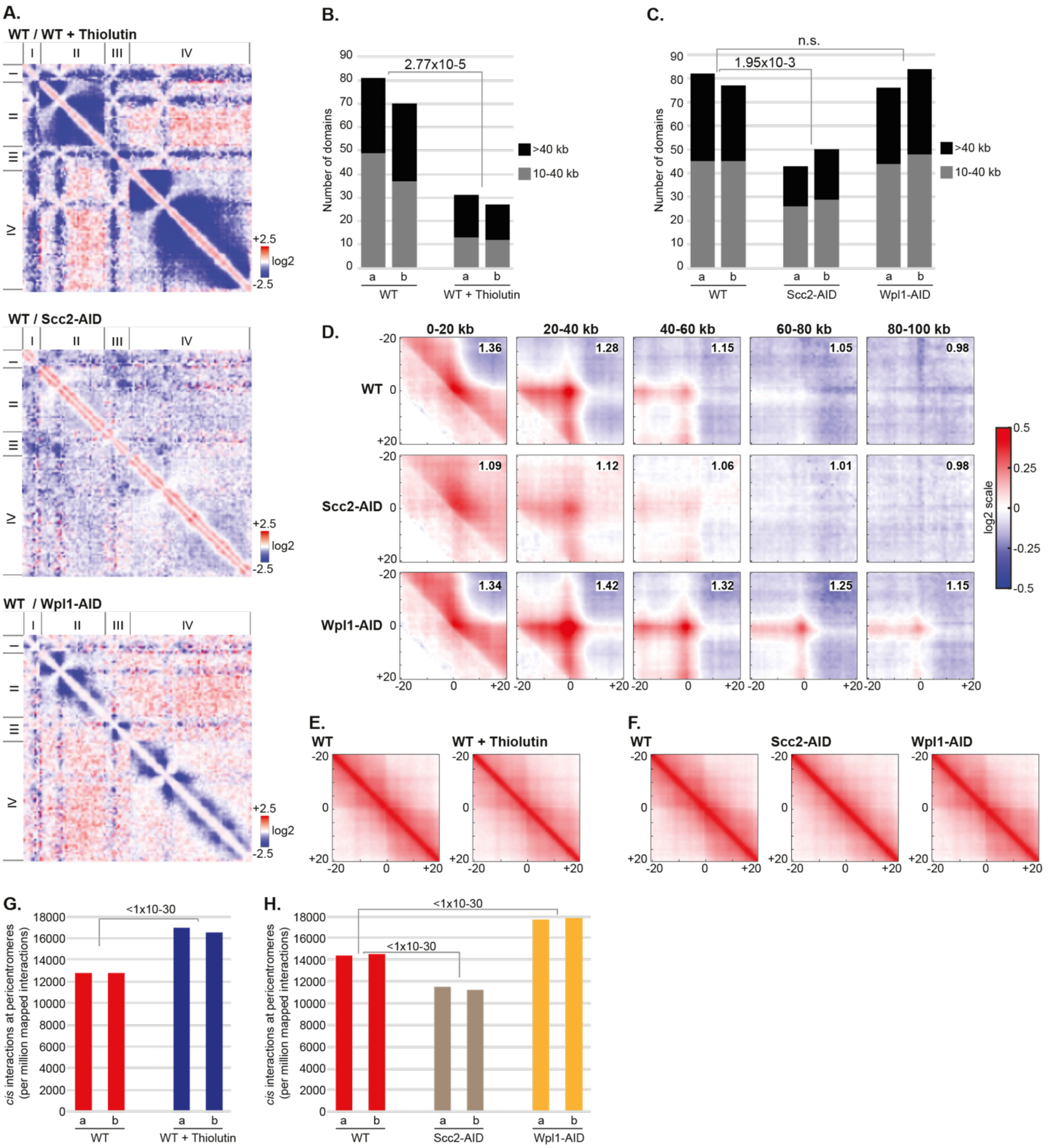

Fig. S3. Transcription inhibition increases chromosome *cis* interactions, reduces the number of domains, but leaves the centromere barrier function unperturbed. **A**. Normalized Hi-C ratio maps (without binning) comparing *cis* and *trans* interaction ratios along and between *S. cerevisiae* chromosomes I-IV in G2/M-arrested wild type (WT) cells with thiolutin-treated, Scc2- or Wpl1-depleted cells. **B**. Number of domains with boundaries at cohesin sites in G2/M-arrested, untreated and thiolutin-treated WT cells. **C**. Number of domains with boundaries at cohesin sites in WT cells, or in cells depleted of Scc2 and Wpl1 (Scc2-AID, Wpl1-AID) in G2/M-arrest. **D**. Aggregate peak analysis centered on interactions between pairs of cohesin sites, separated by

increasing chromosomal distances as indicated on top of the panels. The analysis is based on Hi-C analysis of G2/M-arrested WT cells, and cells depleted for Scc2 or Wpl1 (Scc2-AID, Wpl1-AID) during the G2/M-arrest. The interactions were normalized to pairs of random sites. Log2 color scale on the right-hand side. **E**. Aggregated centromere plots of 40 kb regions spanning each of the 16 S. cerevisiae centromeres in G2/M-arrested, untreated and thiolutin-treated WT cells. **F**. Aggregated centromere plots of 40 kb regions spanning each of the 16 S. cerevisiae centromeres in G2/M-arrested WT cells, and cells depleted for Scc2 or Wpl1 (Scc2-AID, Wpl1-AID) during the G2/M-arrest. **G**. Quantification of *cis* interactions (>10 kb) anchored in pericentromeric regions G2/M-arrested, untreated and thiolutin-treated WT cells. **H**. Quantification of *cis* interactions (>10 kb) anchored in pericentromeric regions G2/M-arrested WT cells, and cells depleted for Scc2 or Wpl1 (Scc2-AID, Wpl1-AID) during the G2/M-arrest. Results from two biological repeats are shown in B-C and G-H. Statistical significance is indicated with p-values from a binominal test in B-C and G-H.

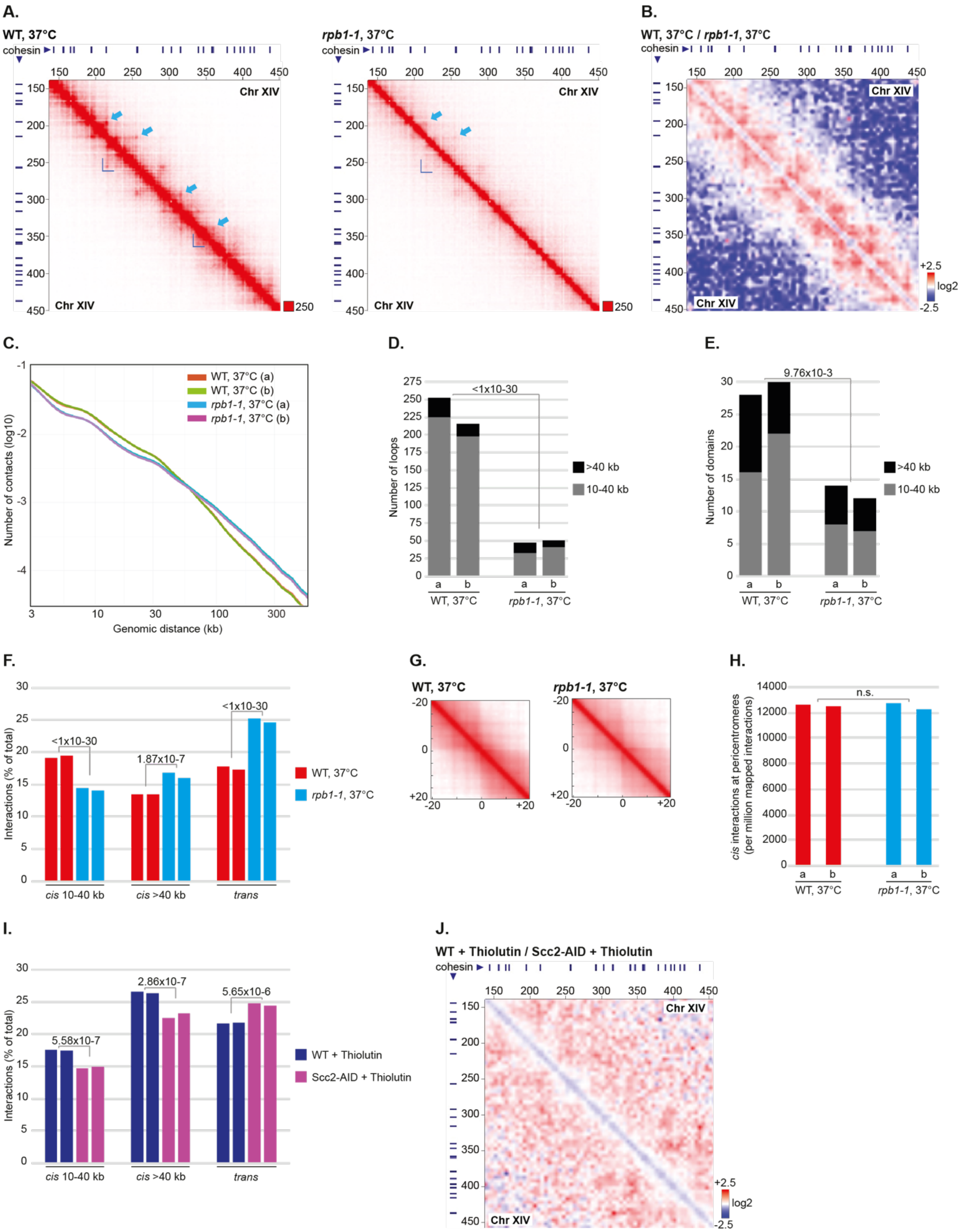

Fig. S4. Inactivation of RNA pol II removes loop extrusion barriers, triggering the formation of long-range *cis* interactions. **A**. Normalized Hi-C contact maps (2 kb binning) showing *cis*

interactions along the arm of chromosome XIV (150-450 kb from left telomere) in G2/M-arrested wild type (WT) and *rpb1-1* cells at 37°C, restrictive temperature for the *rpb1-1* allele. Blue lines on top and to the left of the panels: cohesin binding sites, dark blue L-shape: example of a domain, light blue arrows: examples of loop anchors. **B**. Normalized Hi-C ratio maps (without binning) comparing chromosome *cis* interactions in G2/M-arrested WT and *rpb1-1* cells at 37°C, along the same chromosomal regions as depicted in A). **C**. Contact probability plots as function of genomic distance comparing interactions in G2/M-arrested WT and *rpb1-1* cells at 37°C. **D**. Number of loops anchored at cohesin sites in G2/M-arrested WT and *rpb1-1* cells at 37°C. **E**. Number of domains with boundaries at cohesin sites in G2/M-arrested WT and *rpb1-1* cells at 37°C. **F**. Quantification of *cis* and *trans* interactions in G2/M-arrested WT and *rpb1-1* cells at 37°C. **G**. Aggregated centromere plots of 40 kb regions spanning each of the 16 *S. cerevisiae* centromeres in G2/M-arrested WT and *rpb1-1* cells at 37°C. **H**. Quantification of *cis* interactions (>10 kb) anchored in pericentromeric regions G2/M-arrested WT and *rpb1-1* cells at 37°C. **I**. Quantification of *cis* and *trans* interactions in G2/M-arrested, thiolutin-treated WT and Scc2-depleted cells. **J**. Normalized Hi-C ratio maps (without binning) comparing chromosome *cis* interactions in G2/M-arrested, thiolutin-treated WT and Scc2-depleted cells, along the same chromosomal regions as depicted in A). Results from two biological repeats are shown in D-F and H-I. Statistical significance is indicated with p-values from a binominal test in D-F and H-I.

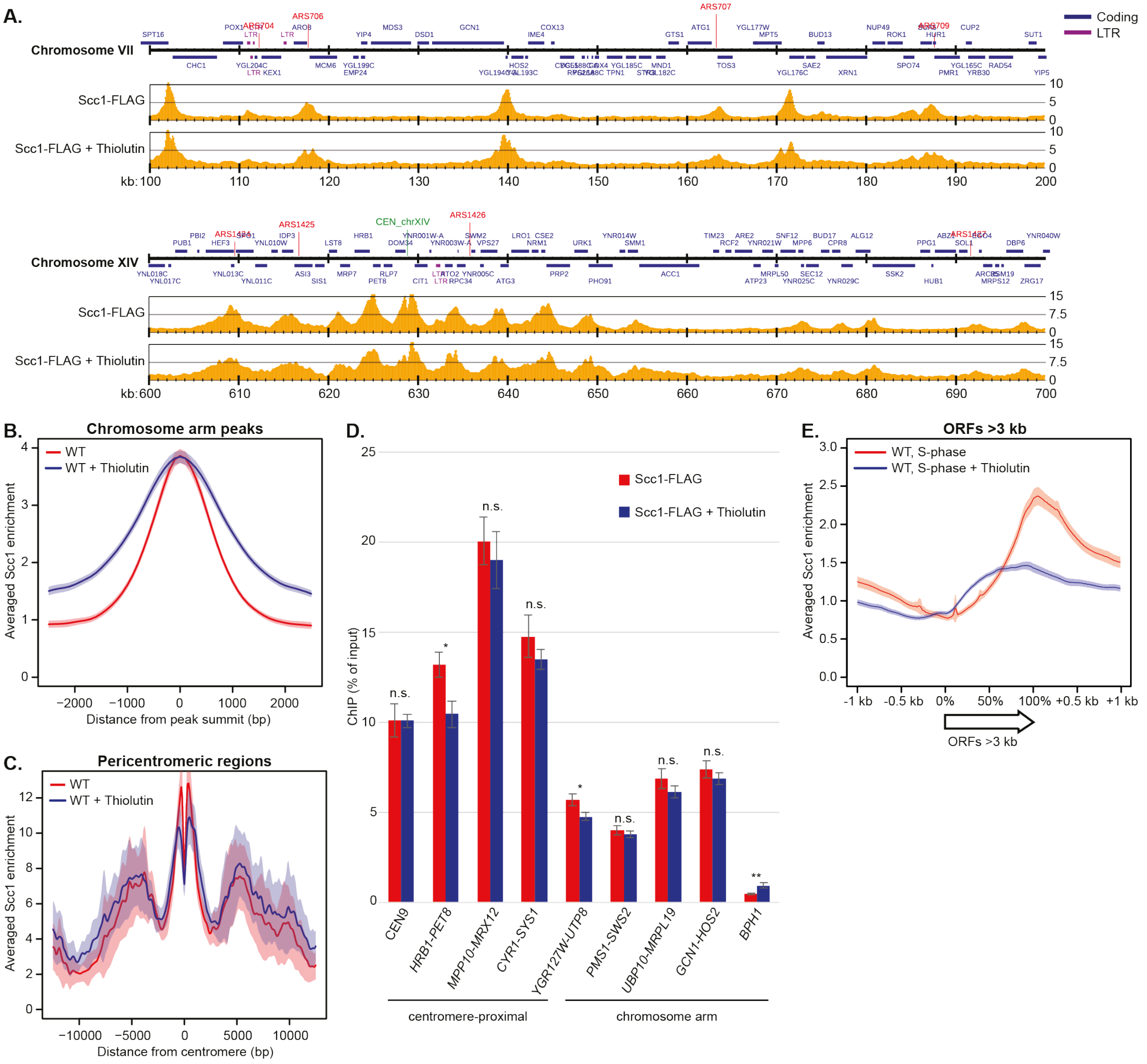

Fig. S5. The effect of transcription inhibition on cohesin chromosomal localization in G2/M and S-phase. A. Scc1-FLAG enrichment on chromosome VII (100-200 kb from left telomere) (two upper maps) and in a region spanning the centromere of chromosome XIV (600-700 kb from left telomere) (two lower maps), based on normalized ChIP-seq analysis of untreated and thiolutin-treated G2/M-arrested wild type (WT) cells. The Y-axis shows fold enrichment of ChIP / input in linear scale, the X-axis shows chromosomal positions. Blue and purple horizontal bars in the uppermost genomic region panel denote coding regions and long terminal repeats (LTRs), respectively, red and green vertical lines indicate replication origins and centromeres, respectively (ARS, CEN). **B**. Averaged Scc1-FLAG enrichment at cohesin sites along chromosome arms, based on the analysis presented in A). The shaded regions indicate the 95% confidence interval. Note that due to the size difference of individual cohesin peaks, the thiolutin-induced splitting of peaks seen in A) is detected as a broadening of the averaged signal. **C**. Averaged Scc1-FLAG enrichment in 25 kb regions spanning each centromere, based on the analysis presented in A). The shaded regions indicate the 95% confidence interval. **D**. Chromosome association of Scc1-FLAG at

centromere IX, at selected intergenic regions flanked by indicated gene-pairs (centromere-proximal or along chromosome arms), and within the *BPH1* ORF, as determined by ChIP-qPCR of samples collected from cells treated as in A). *N=3,* n.s.: $p>0.05$, * : $p\leq0.05$, ** : $p\leq0.01$. **E**. Averaged Scc1-FLAG enrichment in S-phase-arrested cells within ORFs longer than 3 kb, based on the analysis of presented in Fig. 3A. The shaded regions indicate the 95% confidence interval.

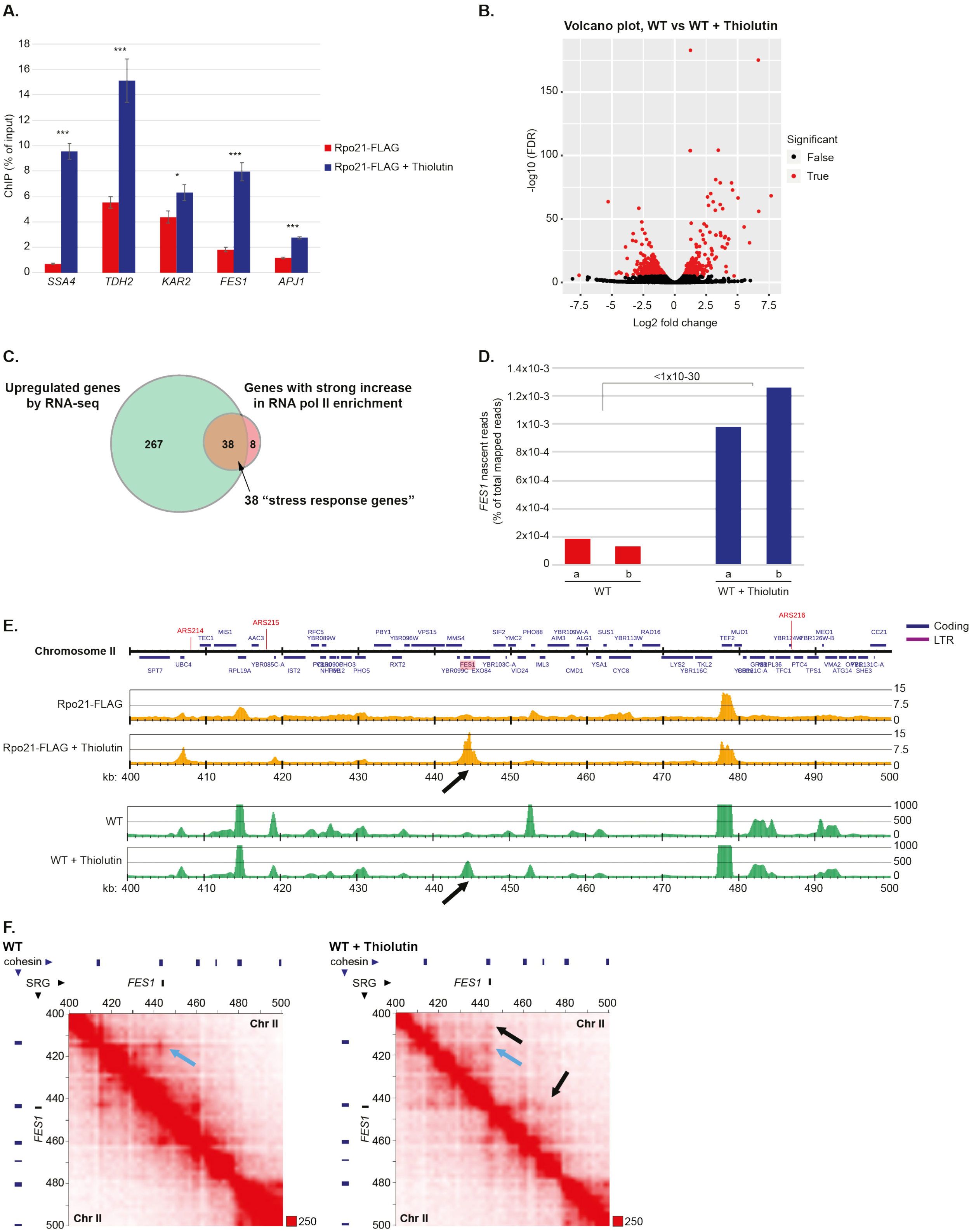

Fig. S6. Thiolutin-induced stress response genes create novel chromosome loop barriers. A. Chromosome association of Rpo21-FLAG in indicated ORFs as revealed by ChIP-qPCR. Note

that *SSA4* and *FES1* are the stress response genes displayed in C) and Fig. 4B. N = 3, n.s.: $p>0.05$, * : $p\leq 0.05$, *** : $p\leq 0.001$. **B**. Volcano plot of differentially expressed genes (DEGs) from spike-in normalized RNA-seq in G2/M-arrested, untreated and thiolutin-treated wild type (WT) cells, in which DEGs (FDR<1x10-5) are highlighted in red. 267 genes out of a total of 6524 *S. cerevisiae* genes were classified as upregulated. **C**. Venn diagram of upregulated DEGs (267 genes) from spike-in normalized RNA-seq, and genes showing normalized ChIP-seq Rpo21-FLAG enrichment above 4.0 in thiolutin treated cells, and below 2.0 in control cells (46 genes). The overlapping 38 genes are defined as stress response genes and used for the analysis in Fig. 4C-E. **D**. Nascent RNA reads in *FES1* ORF, the sole intron-containing stress response gene, as determined by spike-in RNA-seq in G2/M-arrested, untreated and thiolutin-treated WT cells. **E**. Rpo21-FLAG enrichment on chromosome II (400-500 kb from left telomere) in G2/M-arrested, untreated and thiolutin-treated WT cells, based on normalized ChIP-seq analysis (two upper maps). The lower two maps show RNA levels based on spike-in normalized RNA-seq, with the Y-axis denoting spike-in normalized read numbers, with annotations as in Fig. 4A. Black arrows indicate thiolutin-induced Rpo21-FLAG accumulation at, upregulation of, the stress response gene *FES1*. **F**. Normalized Hi-C contact maps (2 kb binning) showing *cis* interactions along the arm of chromosome II (400-500 kb from left telomere) in G2/M-arrested, untreated and thiolutin-treated WT cells. Black arrows indicate barrier formation and *cis* interactions at the *FES1* gene, induced by thiolutin. Blue arrows highlight interactions that are reduced by thiolutin treatment. Blue and black lines on top and to the left of the panels: cohesin binding sites and stress response gene (SRG), respectively.

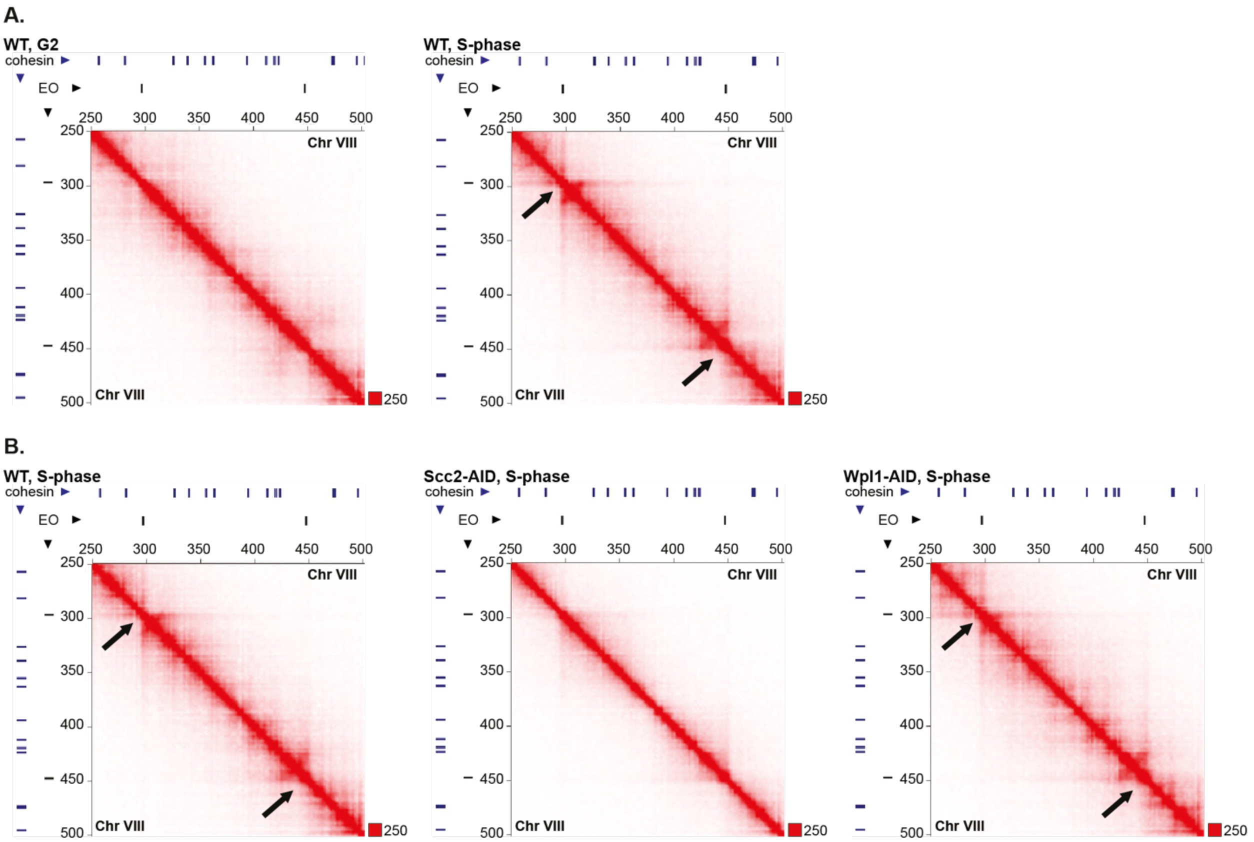

Fig. S7. Additional examples of loop extrusion barriers formed at stalled replication forks. **A**. Normalized Hi-C contact maps (2 kb binning) showing *cis* interactions along the arm of chromosome VIII (250-500 kb from left telomere) in G2/M- and S-phase-arrested wild type (WT) cells. Black arrows highlight loop extrusion boundaries formed at early origins. Blue and black lines on top and to the left of the panels: cohesin binding sites and early firing origin (EO), respectively. **B**. Normalized Hi-C contact maps in S-phase-arrested WT cells, or in cells depleted of Scc2 and Wpl1 (Scc2-AID, Wpl1-AID), as in A).

Table S1. Yeast strains used in this study

All *Saccharomyces cerevisiae* strains are of W303 origin (*ade2-1 trp1-1 can1-100 leu2-3,112 his3-11,15 ura3-1*) *RAD5*, with the modifications listed below.

CB67	*MATa*
CB234	*MATa SCC1-3xFLAG-KAN*
CB1958	*MATa RPO21-3xFLAG-KAN*
CB3060	*MATa ura3-1::ADH1-OsTIR1-9xMYC-URA3 leu2-3,112::tetR'-SSN6-LEU2*
CB3537	*MATa Nat-tTA-tetO7-3xHA-AID*-SCC2 ura3-1::ADH1-OsTIR1-9xMYC-URA3 leu2-3,112::tetR'-SSN6-LEU2*
CB3593	*MATa Nat-tTA-PtetO7-3xHA-AID*-WPL1 ura3-1::ADH1-OsTIR1-9xMYC-URA3 leu2-3,112::tetR'-SSN6-LEU2*
CB3295	*MATa rpb1-1*

The *Schizosaccharomyces pombe* strain used for spike-in normalization in RNA-seq.

CB3416	*h- ade6M216 leu1-32 ura4-D18*

Table S2. ChIP-qPCR primers

Site of amplicon	Sequence 5'- 3'
CEN9, chr. IX, ~356 kb	CACGAATACGAGATACAGGGTAATGAA
	ACAGCTGAAGCTTGCCTCTGTATG
HRB1-PET8 IGR, chr. XIV, pericentromere, ~625 kb	AGACAAGAGGCCTCAGAAGGCTTA
	AACGGCTGGAGAAATGAGAGCGTA
CYR1-SYS1 IGR, chr. X, pericentromere, ~431 kb	ATCTTCAATGTCATCACGTACT
	AAATGGCAAGAACCTTATCTCT
YGR127W-*UTP8* IGR, chr. VII, arm, ~748 kb	CATTCCGGTATCTCTTGTTGGA
	GCGTCTACTGTGGTTACTGATAA
PMS1-SWS2 IGR, chr. XIV, arm, ~476 kb	ATACAGGGAGTGACAACACAAA
	CCACGTTCATATTCTTAATGGCTAAG
UBP10-MRPL19 IGR, chr. XIV, arm, ~292 kb	CCTTCCATCTCCAATAAACTATGCC
	GACCAACCCGTGCTTTAGGAGAGTTA
GCN1-HOS2 IGR, chr. VII, arm, ~140 kb	GCTCCCAGCATTGTAGCTAAT
	ATGATGTGGTGGCAAGTCTC
BPH1 ORF, chr. III, arm, ~181 kb	CGGAAGTGGGTAAGTGCTATT
	ACTACCGGATTCTGTCATGTTT
MPP10-MRX12 IGR, chr. X, pericentromere, ~441 kb	CCCCTTAAAGGACCACTTAAA
	TCAGCACATTCTTCTGGTAT
SSA4 ORF, chr. V, arm, ~366 kb	TAGTAACGGTTCCAGCCTATTTC
	CGGCAGCTGTAGGTTCATTA
TDH2 ORF, chr. X, arm, ~454 kb	CTCTAACGACTACTCCGCTTAC
	CGATGATGTGCTTGTCATCGT
KAR2 ORF, chr. X, arm, ~382 kb	GACTCCTTCGTTGATGGTATCG
	GACAGGCTTCAAGGTCTTCTT
FES1 ORF, chr. II, arm, ~444 kb	CCACTCGACGTGAGAACAAA
	CTATGCAGTCGAGCCCATTTA
MET6 ORF, chr. V, arm, ~341 kb	GCTATGTCCGTCAAGGAATCT
	GTCTCTTGGGAAAGACCATCTC
PTC2 ORF, chr. V, arm, ~337 kb	AGACAAGAGAAGAAGGGTGTTTAG
	GTCCTGAGGTTTCTTCGTAGTG
RPL23B ORF, chr. V, arm, ~397 kb	CCGTCAAAGGTTCTGGTTCT
	TCTCAATTCTGGCTTACCCTTT
ALD5 ORF, chr. V, arm, ~305 kb	CGGTAGAGTTGTTGGAGAAAGA
	CGGCAGCGACCTTCATAATA
VTC1 ORF, chr. V, arm, ~303 kb	GGGTCAGTGCAGGACTATTTAC
	GGACCTGATCCTCTACGTCTAA
RAD51 ORF, chr. V, arm, ~351 kb	TTCTTCCACCACGCGATTAG
	ACACATTCAGCCTCTGGTAAG
APJ1 ORF, chr. XIV, arm, ~482 kb	CAGTTTATGTGGAGGCGTAGTT
	AACAACCAGGCTTCAGTATCTC
MHP1 ORF, chr. X, arm, ~363 kb	GCCAAGTCTCTGCACTCTATT
	CCCAGTTCATGTCGGATCTATT

ARS108, chr. I, pericentromere, early ARS, ~148 kb	TCTGAGTGAAGGGTGGTCTAT
	TGGTGGCGCTGTACATTT
ARS307, chr. III, pericentromere, early ARS, ~109 kb	AGTGATGCGGACATCGTTAATA
	CAGGCAGTTAGGAACTCAACA
ARS418, chr. IV, arm, early ARS, ~555 kb	AGAACATCTTCTCAACGCGAAA
	GGGTTACTCATAAGTGTACCGAAG
ARS920, chr. IX, pericentromere, early ARS, ~357 kb	TTGGTAGGCGTGGAAGATTAC
	TCATTAAGGATACGGCACTGTT
CSL4-PDR16 IGR, chr. XIV, arm, ~216 kb	TTTCTGTAGCGCCTGTAACC
	TTGACCACTGCAAGGAATGA
RPL17A-COY1 IGR, chr. XI, arm, ~110 kb	CGGATAATCCTACGTTCAAAGTATTG
	GAGAGGGAATGAGTAACGATTGA

***** IGR: intergenic region, ORF: open reading frame

Table S3. Hi-C sequencing statistics (separate .xlsx file)

Table S4. ChIP-seq and RNA-seq statistics (separate .xlsx file)

Table S5. Differentially Expressed Genes (separate .xlsx file)

Table S6. ChIP-seq ChIP-qPCR normalization (separate .xlsx file)
